# Age-stratified risk of major systemic complications following unicompartmental versus total knee arthroplasty: a nationwide cohort study

**DOI:** 10.1186/s43019-026-00330-8

**Published:** 2026-07-31

**Authors:** Manabu Kawata, Shotaro Aso, Kohei Kawaguchi, Ryota Yamagami, Kenichi Kono, Shuji Taketomi, Hiroki Matsui, Kiyohide Fushimi, Hideo Yasunaga, Sakae Tanaka

**Affiliations:** 1 Department of Orthopaedic Surgery, Tokyo Metropolitan Institute for Geriatrics and Gerontology, Tokyo, Japan; 2 Department of Health Services Research, Graduate School of Medicine, The University of Tokyo, Tokyo, Japan; 3 Department of Orthopaedic Surgery, Faculty of Medicine, The University of Tokyo, Tokyo, Japan; 4 Department of Clinical Epidemiology and Health Economics, School of Public Health, The University of Tokyo, Tokyo, Japan; 5 Department of Health Policy and Informatics, Institute of Science Tokyo Graduate School, Tokyo, Japan

**Keywords:** Total knee arthroplasty, Unicompartmental knee arthroplasty, Postoperative complication, Propensity score, Diagnosis Procedure Combination database

## Abstract

**Background:**

The optimal surgical approach for knee arthroplasty remains under debate regarding reducing systemic complications. Although unicompartmental knee arthroplasty (UKA) is generally considered less invasive than total knee arthroplasty (TKA), it remains unclear whether this advantage persists across age groups. This study aimed to compare the risk of major systemic complications between UKA and TKA across age categories using a large-scale nationwide database.

**Methods:**

Patients who underwent UKA or TKA between July 2010 and March 2022 were identified from the Diagnosis Procedure Combination database in Japan. The primary outcome was a composite of postoperative in-hospital death and major systemic complications requiring additional interventions. Rates of postoperative red blood cell (RBC) transfusion were also evaluated as a secondary outcome. Stabilized inverse probability of treatment weighting (IPTW) using propensity scores was applied to compare outcomes between the groups.

**Results:**

The cohort included 36,235 UKA and 322,424 TKA cases. After stabilized IPTW adjustment, the composite outcome occurred less frequently after UKA than after TKA (risk ratio [RR] 0.65; 95% confidence interval [CI] 0.50–0.85; *p* = 0.001). The RBC transfusion rate was also reduced in the UKA group (RR, 0.09; 95% CI 0.08–0.11; *p* < 0.001). In subgroup analyses stratified by age (≤ 79 and ≥ 80 years), UKA was associated with a lower incidence of the composite outcome compared with TKA in patients aged ≤ 79 years (RR 0.48; 95% CI 0.34–0.68; *p* < 0.001), whereas no significant difference between UKA and TKA was observed among patients aged ≥ 80 years (RR 0.92; 95% CI 0.62–1.35; *p* = 0.670). The rate of RBC transfusion was lower in the UKA group across age groups (for ≤ 79 years, RR 0.07; 95% CI 0.05–0.09; *p* < 0.001; for ≥ 80 years, RR 0.12; 95% CI 0.10–0.14; *p* < 0.001).

**Conclusions:**

RBC transfusion rates were consistently lower following UKA across age groups. While UKA was associated with fewer major systemic complications than TKA in patients aged ≤ 79 years, no such difference was observed in patients aged ≥ 80 years. In very elderly patients, careful perioperative risk assessment remains essential when considering UKA, similar to that for TKA.

**Supplementary Information:**

The online version contains supplementary material available at 10.1186/s43019-026-00330-8.

## Introduction

Total knee arthroplasty (TKA) is the standard surgical procedure used to treat various knee diseases, including osteoarthritis, osteonecrosis, and rheumatoid arthritis, primarily in middle-aged and older patients [1–5]. Unicompartmental knee arthroplasty (UKA) is an effective surgical treatment for unicompartmental knee conditions, including osteoarthritis and osteonecrosis [1, 6–10]. The demand for knee arthroplasty has been rising steadily in aging and increasingly overweight populations [2, 11, 12], and the utilization of UKA has expanded across a wide range of age groups, including older patients [4].

Compared with TKA, UKA offers several advantages, such as preservation of soft tissues and bone, reduced blood loss, fewer perioperative complications, faster recovery, lower cost, superior clinical outcomes, and greater patient satisfaction [9, 13–24]. However, UKA is also associated with higher rates of revision and periprosthetic fracture compared with TKA [6, 13, 15, 18, 23, 25, 26]. Among the reported benefits of UKA, lower rates of postoperative major systemic complications and in-hospital mortality are particularly important because these outcomes represent some of the most serious outcomes following elective surgery [13, 18]. However, evidence directly comparing these outcomes between UKA and TKA across different age groups remains limited. Therefore, we conducted a nationwide retrospective cohort study using a large administrative claims database to compare the occurrence of rare but clinically important major systemic complications, including cardiovascular, respiratory, thromboembolic, cerebrovascular, renal, and gastrointestinal complications, between UKA and TKA across age-stratified subgroups, with sufficient statistical power to detect such outcomes.

The purpose of this study was to compare the risk of postoperative major systemic complications requiring additional procedures or interventions, in-hospital mortality, and the rate of transfusion between UKA and TKA across different age categories. We hypothesized that the association between procedure type and postoperative complications would vary across age groups.

## Materials and methods

### Data source

Inpatient data from July 2010 to March 2022 were obtained from the Japanese Diagnosis Procedure Combination (DPC) database. The DPC database includes discharge abstracts and administrative claims from participating hospitals. Detailed descriptions of the database have been provided previously [4, 27–33]. Participation in the DPC database is mandatory for all academic hospitals in Japan and voluntary for community hospitals. As of 2021, the database comprised data from 1171 hospitals and covered 7.16 million patients, representing approximately half of all inpatient admissions to acute care hospitals in Japan, which are typically responsible for performing major surgical procedures, including knee arthroplasty. Diagnoses and surgical or interventional procedures are recorded by attending physicians on the basis of medical records to ensure accurate reimbursement claims. A previous validation study demonstrated high sensitivity and specificity of diagnostic and procedural coding in the DPC database [34].

### Study subjects

Diagnoses were coded in accordance with the International Classification of Diseases, 10th Revision (ICD-10). Patients who underwent knee arthroplasty within 14 days of hospital admission were included. The exclusion criteria were as follows: bilateral knee arthroplasty during a single admission; procedures other than TKA or UKA, or unknown procedure types; and primary diagnoses other than osteoarthritis (ICD-10 code: M17), osteonecrosis (M87), or rheumatoid arthritis (M05 and M06).

The following variables were extracted from the DPC: dates of admission and discharge, sex, age, body mass index (BMI), smoking status, primary diagnosis, preexisting comorbidities, surgical procedures, surgical materials (including implants), anesthesia type, transfusions and medications administered during hospitalization, and in-hospital complications. Definitions of preoperative comorbidities are provided in Supplementary Table 1.

### Outcomes

To ensure diagnostic specificity and focus on rare but clinically significant major outcomes, the primary outcome was defined as a composite of postoperative in-hospital death and major systemic complications requiring additional procedures or interventions. These complications included cardiac arrest, acute coronary events, heart failure, aortic aneurysm or dissection, respiratory failure requiring mechanical ventilation (respiratory failure), pulmonary embolism, cerebrovascular events, renal failure requiring hemodialysis, and gastrointestinal bleeding or peptic ulcer perforation (gastrointestinal bleeding/perforation). Detailed definitions of each complication are provided in Supplementary Table 2. Secondary outcomes were the individual components of the composite outcome, and postoperative RBC transfusion.

### Statistical analysis

Categorical variables were presented as frequencies and percentages, and chi-squared tests were used to compare unadjusted outcomes.

To compare the primary outcome between the UKA and TKA groups, stabilized inverse probability of treatment weighting (IPTW) based on propensity scores was applied to adjust for baseline differences [35–37]. IPTW helps balance measured confounders while preserving the sample size. Propensity scores were estimated using logistic regression with the following covariates: sex, age group, BMI category, smoking status, primary diagnosis, comorbidities, steroid use, immunosuppressant use, and anesthesia type. Covariate balance was assessed using absolute standardized differences, with values < 0.1 indicating acceptable balance. Subgroup analyses were conducted according to age group.

All statistical analyses were two-sided, with a significance threshold of *p* < 0.05. Analyses were performed using STATA/SE version 18.0 (StataCorp, College Station, TX, USA).

## Results

**Fig. 1:**
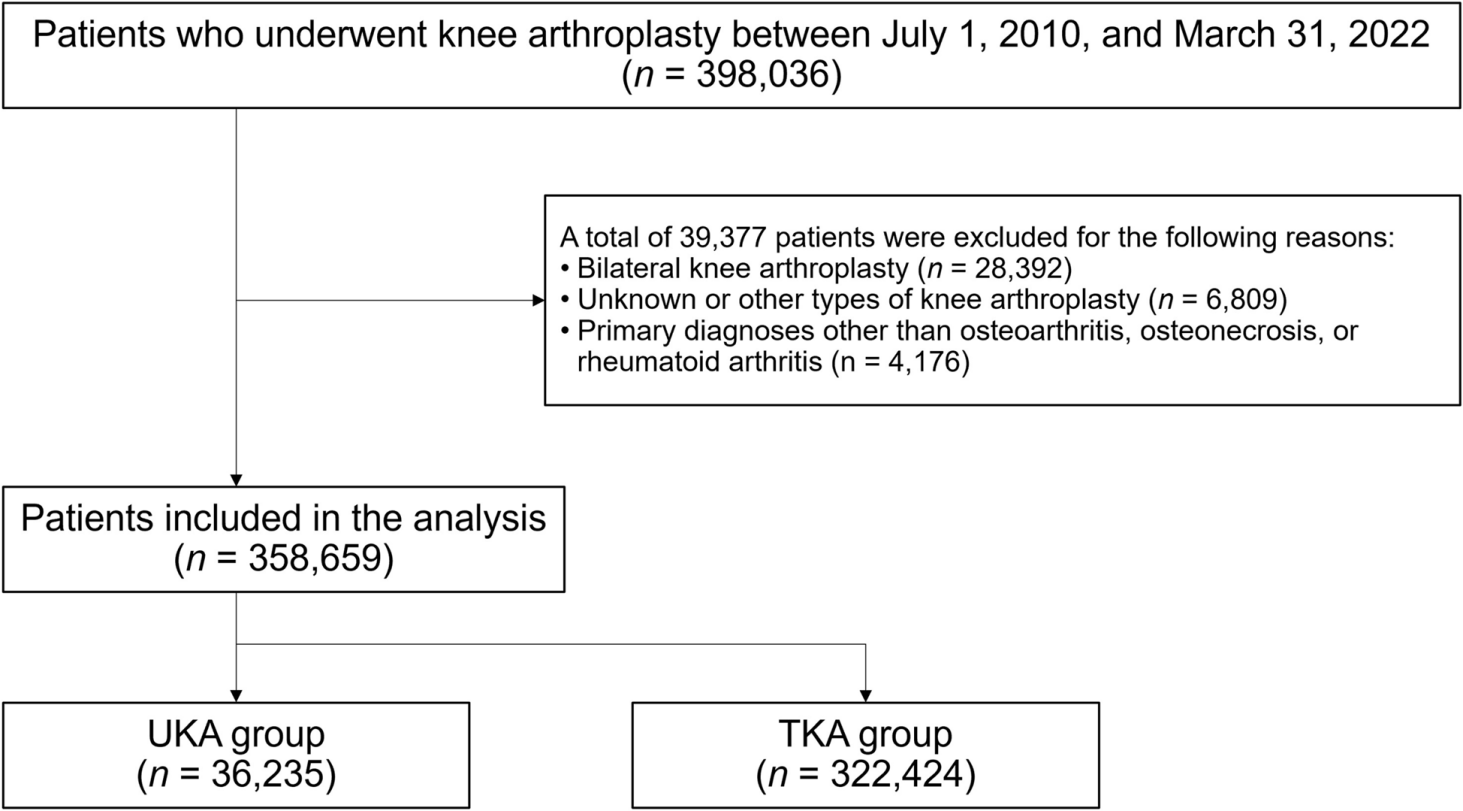
Flow diagram of the patient selection process. *UKA* unicompartmental knee arthroplasty, *TKA* total knee arthroplasty

Figure 1 summarizes the patient selection process. Of the 398,036 patients who underwent knee arthroplasty during the study period, 39,377 were excluded, resulting in 36,235 patients in the UKA group and 322,424 in the TKA group. The median postoperative length of stay was 23 days (interquartile range, 19–31 days) in the TKA cohort and 19 days (14–25 days) in the UKA cohort.

Table 1 presents the patients’ baseline characteristics before and after stabilized IPTW. Prior to weighting, baseline characteristics differed between the UKA and TKA groups in sex, BMI category, smoking status, primary diagnosis, steroid use, immunosuppressant use, and anesthesia type. Table 2 presents unadjusted comparisons of postoperative complications between UKA and TKA. Compared with the TKA group, the UKA group exhibited lower incidences of acute coronary events, heart failure, respiratory failure, pulmonary embolism, gastrointestinal bleeding/perforation, and overall major complications, as well as a lower rate of RBC transfusion.

**Table 1:** Baseline characteristics of patients who underwent UKA and TKA before and after stabilized inverse probability of treatment weighting

	Baseline			After IPTW^c^ adjustment		
	UKA^a^ *n* (%)	TKA^b^ *n* (%)	ASD	UKA (%)	TKA (%)	ASD^d^
Overall patient numbers	36,235	322,424		34,274	301,067	
Male sex	9477 (26.2)	64,025 (19.9)	0.150^*^	(20.7)	(19.9)	0.018
Age (years)			0.086			0.027
≤ 59	1471 (4.1)	12,591 (3.9)		(3.5)	(3.9)	
60–69	7181 (19.8)	58,589 (18.2)		(18.2)	(18.2)	
70–79	18,541 (51.2)	159,522 (49.5)		(49.3)	(49.7)	
80–89	8775 (24.2)	89,769 (27.8)		(28.4)	(27.6)	
≥ 90	267 (0.7)	1953 (0.6)		(0.6)	(0.6)	
Body mass index (kg/m^2^)			0.234^*^			0.011
< 25.0	18,638 (51.4)	139,873 (43.4)		(45.0)	(44.7)	
25.0–29.9	14,196 (39.2)	130,494 (40.5)		(40.5)	(40.5)	
30.0–34.9	2782 (7.7)	40,597 (12.6)		(11.9)	(12.1)	
≥ 35.0	398 (1.1)	9268 (2.9)		(2.6)	(2.7)	
Unknown	221 (0.6)	2192 (0.7)		(–)	(–)	
Smoking status			0.108^*^			0.022
Smoker	5714 (15.8)	39,461 (12.2)		(14.3)	(13.5)	
Non-smoker	28,119 (77.6)	263,327 (81.7)		(85.7)	(86.5)	
Unknown	2402 (6.6)	19,636 (6.1)		(–)	(–)	
Primary diagnosis			0.643^*^			0.078
Osteoarthritis	29,733 (82.1)	301,849 (93.6)		(90.8)	(92.5)	
Osteonecrosis	6466 (17.8)	5239 (1.6)		(3.3)	(3.3)	
Rheumatoid arthritis	36 (0.1)	15,336 (4.8)		(5.9)	(4.2)	
Diabetes mellitus	4737 (13.1)	47,802 (14.8)	0.051	(14.8)	(14.7)	0.004
Dyslipidemia	4706 (13.0)	40,327 (12.5)	0.014	(12.6)	(12.7)	0.002
Hypertension	8846 (24.4)	85,209 (26.4)	0.046	(26.8)	(26.4)	0.009
Ischemic heart disease	2214 (6.1)	22,987 (7.1)	0.041	(6.8)	(7.0)	0.007
Valve disorder	903 (2.5)	10,642 (3.3)	0.048	(3.2)	(3.3)	0.007
Atrial fibrillation/flutter	922 (2.5)	9625 (3.0)	0.027	(2.8)	(2.9)	0.009
Heart failure	777 (2.1)	10,386 (3.2)	0.067	(3.0)	(3.1)	0.007
Cerebrovascular disease	1291 (3.6)	12,581 (3.9)	0.018	(3.8)	(3.9)	0.006
Malignancy	685 (1.9)	7005 (2.2)	0.020	(2.0)	(2.2)	0.008
Hypothyroidism	317 (0.9)	2978 (0.9)	0.005	(0.9)	(0.9)	0.001
Hyperthyroidism	122 (0.3)	1191 (0.4)	0.006	(0.4)	(0.4)	< 0.001
Dementia	330 (0.9)	3229 (1.0)	0.009	(1.0)	(1.0)	< 0.001
Parkinson’s disease	181 (0.5)	2060 (0.6)	0.019	(0.6)	(0.6)	0.001
Epilepsy	144 (0.4)	1742 (0.5)	0.021	(0.5)	(0.5)	< 0.001
Chronic lung disease	1022 (2.8)	9947 (3.1)	0.016	(2.9)	(3.1)	0.009
Noninfective enteritis	33 (0.1)	454 (0.1)	0.015	(0.1)	(0.1)	< 0.001
Hepatic dysfunction	294 (0.8)	2963 (0.9)	0.012	(0.8)	(0.9)	0.008
Renal dysfunction	658 (1.8)	7488 (2.3)	0.036	(2.6)	(2.3)	0.023
Steroids	687 (1.9)	15,324 (4.8)	0.160^*^	(6.5)	(4.4)	0.090
Immunosuppressants	188 (0.5)	13,057 (4.0)	0.238^*^	(5.3)	(3.7)	0.081
Anesthesia type			0.127^*^			0.016
General	31,054 (85.7)	288,356 (89.4)		(89.4)	(89.0)	
Spinal	4332 (12.0)	26,268 (8.1)		(8.5)	(8.6)	
Other types	849 (2.3)	7800 (2.4)		(2.2)	(2.4)	

^a^Unicompartmental knee arthroplasty

^b^Total knee arthroplasty

^c^Inverse probability of treatment weighting

^d^Absolute standardized difference

^*^ASD ≥ 0.1 indicates covariate imbalance

**Table 2:** Unadjusted comparisons of postoperative complications between UKA and TKA

Complication	UKA^a^ (*n* = 36,235) *n* (%)	TKA^b^ (*n* = 322,424) *n* (%)	*p*-Value^c^
In-hospital death	14 (0.04)	173 (0.05)	0.235
Cardiac arrest	10 (0.03)	136 (0.04)	0.192
Acute coronary event	8 (0.02)	154 (0.05)	0.029^*^
Heart failure	11 (0.03)	221 (0.07)	0.007^*^
Aortic aneurysm or dissection	2 (< 0.01)	13 (< 0.01)	0.678
Respiratory failure^d^	34 (0.09)	431 (0.13)	0.046^*^
Pulmonary embolism	16 (0.04)	301 (0.09)	0.003^*^
Cerebrovascular event	1 (< 0.01)	39 (0.01)	0.111
Renal failure^e^	1 (< 0.01)	27 (< 0.01)	0.251
Gastrointestinal bleeding/perforation^f^	21 (0.06)	306 (0.10)	0.027^*^
Overall major complications	89 (0.25)	1458 (0.45)	< 0.001^*^
Red blood cell transfusion	244 (0.67)	26,265 (8.15)	< 0.001^*^

^a^Unicompartmental knee arthroplasty

^b^Total knee arthroplasty

^c^UKA versus TKA

^d^respiratory failure requiring mechanical ventilation

^e^Renal failure requiring hemodialysis

^f^Gastrointestinal bleeding or peptic ulcer perforation

^*^p < 0.05 indicates statistical significance

To evaluate the age-stratified risk of major systemic complications following UKA versus TKA, we first performed adjusted comparisons between UKA and TKA as well as across age categories using multivariable logistic regression analysis, incorporating all variables presented in Table 1 (results presented in Table 3). The incidence of overall major complications was lower in the UKA group than in the TKA group (risk ratio [RR] 0.57; 95% confidence interval [CI] 0.45–0.72; *p* < 0.001). Among patients aged ≥ 80 years, the incidence of overall major complications was significantly higher than that among patients aged 70–79 years (for 80–89 years, OR 1.32; 95% CI 1.18–1.49; *p* < 0.001; for ≥ 90 years, OR 2.05; 95% CI 1.29–3.24; *p* = 0.002). On the basis of these findings, patients were stratified into those aged ≥ 80 years and ≤ 79 years for subsequent comparisons.

**Table 3:** Multivariable logistic regression analysis for overall major systemic complications (*n* = 334,704)

	Odds ratio	95% confidence interval	*p*-Value
Type of procedure			
UKA^a^	0.57	0.45–0.72	< 0.001^*^
TKA^b^	Reference		
Age (years)			
≤ 59	0.84	0.61–1.15	0.277
60–69	0.79	0.67–0.93	0.006^*^
70–79	Reference		
80–89	1.32	1.18–1.49	< 0.001^*^
≥ 90	2.05	1.29–3.24	0.002^*^
Male sex	1.34	1.18–1.53	< 0.001^*^
Body mass index (kg/m^2^)			
< 25.0	Reference		
25.0–29.9	0.82	0.73–0.93	0.001^*^
30.0–34.9	1.01	0.85–1.19	0.951
≥ 35.0	1.61	1.22–2.12	0.001^*^
Smoking status			
Smoker	0.99	0.84–1.16	0.910
Non-smoker	Reference		
Primary diagnosis			
Osteoarthritis	Reference		
Osteonecrosis	0.83	0.57–1.21	0.323
Rheumatoid arthritis	0.72	0.52–0.99	0.044^*^
Diabetes mellitus	1.24	1.08–1.42	0.002^*^
Dyslipidemia	1.04	0.89–1.21	0.645
Hypertension	1.12	1.00–1.26	0.050
Ischemic heart disease	2.13	1.84–2.46	< 0.001^*^
Valve disorder	1.81	1.47–2.21	< 0.001^*^
Atrial fibrillation/flutter	2.25	1.85–2.73	< 0.001^*^
Heart failure	1.85	1.53–2.25	< 0.001^*^
Cerebrovascular disease	1.27	1.01–1.59	0.039^*^
Malignancy	1.57	1.20–2.06	0.001^*^
Hypothyroidism	0.90	0.53–1.53	0.702
Hyperthyroidism	0.47	0.15–1.48	0.200
Dementia	1.18	0.76–1.82	0.456
Parkinson’s disease	1.46	0.84–2.54	0.176
Epilepsy	2.07	1.26–3.41	0.004^*^
Chronic lung disease	1.57	1.24–1.97	< 0.001^*^
Noninfective enteritis	1.33	0.42–4.14	0.628
Hepatic dysfunction	1.52	0.98–2.34	0.061
Renal dysfunction	2.96	2.44–3.60	< 0.001^*^
Steroids	2.47	2.01–3.03	< 0.001^*^
Immunosuppressants	0.79	0.57–1.10	0.166
Anesthesia type			
General	Reference		
Spinal	0.74	0.59–0.92	0.007^*^
Other types	1.24	0.91–1.70	0.165

^a^Unicompartmental knee arthroplasty

^b^Total knee arthroplasty

^*^p < 0.05 indicates statistical significance

The results of the age-stratified subgroup analyses are presented in Table 4. Among patients aged ≤ 79 years, the UKA group had significantly lower incidences of in-hospital death, heart failure, respiratory failure, pulmonary embolism, gastrointestinal bleeding/perforation, and overall major complications, and a lower rate of RBC transfusion, compared with the TKA group. In contrast, among patients aged ≥ 80 years, no statistically significant differences were observed between the groups for any of the evaluated major systemic complications, although the UKA group showed a lower rate of RBC transfusion (*p* < 0.001).

**Table 4:** Unadjusted subgroup analyses of postoperative complications by age group between the UKA and TKA groups

Complication	≤ 79 years			≥ 80 years		
	UKA^a^ (*n* = 27,193) *n* (%)	TKA^b^ (*n* = 230,702) *n* (%)	*p*-Value^c^	UKA (*n* = 9042) *n* (%)	TKA (*n* = 91,722) *n* (%)	*p*-Value^a^
In-hospital death	4 (0.02)	93 (0.04)	0.039^*^	10 (0.11)	80 (0.09)	0.478
Cardiac arrest	5 (0.02)	77 (0.03)	0.190	5 (0.06)	59 (0.06)	0.745
Acute coronary event	6 (0.02)	99 (0.04)	0.107	2 (0.02)	55 (0.06)	0.149
Heart failure	6 (0.02)	130 (0.06)	0.020^*^	5 (0.06)	91 (0.10)	0.197
Aortic aneurysm or dissection	1 (< 0.01)	3 (< 0.01)	0.347	1 (0.01)	10 (0.01)	0.989
Respiratory failure^d^	17 (0.06)	274 (0.12)	0.009^*^	17 (0.19)	157 (0.17)	0.713
Pulmonary embolism	12 (0.04)	201 (0.09)	0.020^*^	4 (0.04)	100 (0.11)	0.067
Cerebrovascular event	0 (0.00)	23 (0.01)	0.100	1 (0.01)	16 (0.02)	0.656
Renal failure^e^	1 (< 0.01)	20 (< 0.01)	0.388	0 (0.00)	7 (< 0.01)	0.406
Gastrointestinal bleeding/perforation^f^	11 (0.04)	199 (0.09)	0.012^*^	10 (0.11)	107 (0.12)	0.872
Overall major complications	48 (0.18)	915 (0.40)	< 0.001^*^	41 (0.45)	543 (0.59)	0.098
Red blood cell transfusion	99 (0.36)	13,856 (6.01)	< 0.001^*^	145 (1.60)	12,409 (13.53)	< 0.001^*^

^a^Unicompartmental knee arthroplasty

^b^Total knee arthroplasty

^c^UKA versus TKA

^d^respiratory failure requiring mechanical ventilation

^e^Renal failure requiring hemodialysis

^f^Gastrointestinal bleeding or peptic ulcer perforation

^*^p < 0.05 indicates statistical significance

After applying stabilized IPTW, baseline covariates were well balanced between the groups (Table 1). Table 5 presents the IPTW-adjusted proportions of postoperative complications between the UKA and TKA groups in the overall cohort and by age group. In addition to overall major complications and RBC transfusion, analyses were also performed for individual outcome components that showed significant differences in the unadjusted analyses, namely, in-hospital death, acute coronary events, heart failure, respiratory failure, pulmonary embolism, and gastrointestinal bleeding/perforation. In the overall cohort, the incidences of pulmonary embolism (RR 0.53; 95% CI 0.29–0.94; *p* = 0.031) and overall major complications (RR 0.65; 95% CI 0.50–0.85; *p* = 0.001) were lower in the UKA group than in the TKA group. The rate of RBC transfusion was also reduced in the UKA group (RR 0.09; 95% CI 0.08–0.11; *p* < 0.001).

**Table 5:** Adjusted comparisons of postoperative complications between the UKA and TKA groups after stabilized inverse probability of treatment weighting

Complication	UKA^a^ (%)	TKA^b^ (%)	Risk ratio^c^	95% confidence interval	*p*-Value^d^
In-hospital death	0.06	0.05	1.12	0.54–2.33	0.760
Age subgroups					
≤ 79 years	0.01	0.04	0.36	0.12–1.06	0.065
≥ 80 years	0.17	0.09	1.92	0.81–4.54	0.139
Acute coronary event	0.02	0.05	0.50	0.23–1.12	0.093
Age subgroups					
≤ 79 years	0.02	0.04	0.49	0.19–1.23	0.128
≥ 80 years	0.03	0.06	0.53	0.12–2.24	0.387
Heart failure	0.04	0.07	0.55	0.28–1.06	0.076
Age subgroups					
≤ 79 years	0.02	0.06	0.37	0.15–0.92	0.032^*^
≥ 80 years	0.08	0.10	0.78	0.31–1.96	0.595
Respiratory failure^e^	0.11	0.13	0.82	0.51–1.34	0.433
Age subgroups					
≤ 79 years	0.05	0.11	0.47	0.24–0.89	0.022^*^
≥ 80 years	0.23	0.17	1.41	0.73–2.74	0.305
Pulmonary embolism	0.05	0.09	0.53	0.29–0.94	0.031^*^
Age subgroups					
≤ 79 years	0.05	0.09	0.49	0.25–0.96	0.038^*^
≥ 80 years	0.06	0.11	0.59	0.20–1.77	0.346
Gastrointestinal bleeding/perforation^f^	0.07	0.09	0.76	0.45–1.27	0.293
Age subgroups					
≤ 79 years	0.06	0.08	0.72	0.36–1.42	0.338
≥ 80 years	0.10	0.12	0.82	0.38–1.80	0.627
Overall major complications	0.29	0.45	0.65	0.50–0.85	0.001^*^
Age subgroups					
≤ 79 years	0.19	0.39	0.48	0.34–0.68	< 0.001^*^
≥ 80 years	0.54	0.59	0.92	0.62–1.35	0.670
Red blood cell transfusion	0.75	8.05	0.09	0.08–0.11	< 0.001^*^
Age subgroups					
≤ 79 years	0.41	5.92	0.07	0.05–0.09	< 0.001^*^
≥ 80 years	1.60	13.50	0.12	0.10–0.14	< 0.001^*^

^a^Unicompartmental knee arthroplasty

^b^Total knee arthroplasty

^c^Risk ratio of UKA relative to TKA

^d^UKA versus TKA

^e^Respiratory failure requiring mechanical ventilation

^f^Gastrointestinal bleeding or peptic ulcer perforation

^*^p < 0.05 indicates statistical significance

Among patients aged ≤ 79 years, the UKA group had a significantly lower risk of overall major complications compared with the TKA group (RR 0.48; 95% CI 0.34–0.68; *p* < 0.001). In contrast, no significant difference in the composite outcome was observed in patients aged ≥ 80 years (RR 0.92; 95% CI 0.62–1.35; *p* = 0.670). For individual outcome components in patients aged ≤ 79 years, the UKA group had significantly lower risks of heart failure (RR 0.37; 95% CI 0.15–0.92; *p* = 0.032), respiratory failure (RR 0.47; 95% CI 0.24–0.89; *p* = 0.022), pulmonary embolism (RR 0.49; 95% CI 0.25–0.96; *p* = 0.038), and RBC transfusion (RR 0.07; 95% CI 0.05–0.09; *p* < 0.001). In-hospital mortality was lower in the UKA group than in the TKA group (RR 0.36; 95% CI 0.12–1.06; *p* = 0.065), although the difference did not reach statistical significance. No statistically significant differences were observed in the ≥ 80-year cohort for any of the evaluated major systemic complications, whereas the rate of RBC transfusion (RR 0.12; 95% CI 0.10–0.14; *p* < 0.001) was lower in the UKA group.

## Discussion

This study compared the occurrence of rare but clinically significant major systemic complications and the proportion of RBC transfusion following UKA and TKA in a nationwide inpatient cohort. Overall major systemic complications, pulmonary embolism, and RBC transfusion were less frequent after UKA than after TKA in the overall cohort. Importantly, the associations between procedure type and postoperative complications differed according to age, indicating a potential effect modification by age. In patients aged ≤ 79 years, UKA was associated with significantly lower risks of overall major systemic complications, heart failure, respiratory failure, pulmonary embolism, and RBC transfusion. In contrast, among patients aged ≥ 80 years, no such advantage of UKA was observed for any of the evaluated major systemic complications, whereas the rate of RBC transfusion was lower in the UKA group.

Previous studies have reported lower rates of complications, mortality, and transfusion following UKA compared with TKA [13, 18, 22–24], and the present study, which focused on more severe complications, demonstrated similar findings. The absolute incidence of the composite outcome of in-hospital death and major systemic complications requiring additional interventions was low in both groups. However, these complications represent clinically important adverse events in elective knee arthroplasty, because patients are likely to rate mortality and major systemic complications as the worst possible outcomes [13]. Furthermore, because knee arthroplasty is a commonly performed procedure, even small absolute differences in incidence of complications may affect a substantial number of patients at the population level [13, 38]. However, it remains unclear whether this advantage is consistent across age groups. One prior study examined early medical complications in a population aged ≥ 85 years, comparing 30 UKA cases with 90 TKA cases, and reported fewer complications in the UKA group [39]. However, the study was limited by its single-center design, small sample size, and reliance on age matching alone. Conversely, the present study used a large-scale nationwide database and applied stabilized IPTW to achieve covariate balance between the UKA and TKA groups. The results demonstrated that patients aged ≤ 79 years experienced significantly lower risks of major systemic complications, as well as a lower rate of RBC transfusion, after UKA than after TKA. Additionally, UKA has been associated with higher patient satisfaction and superior functional outcomes compared with TKA in younger patients [40]. Our findings reinforce the idea that, particularly in younger individuals, UKA may be a favorable surgical option not only in terms of functional advantages but also regarding perioperative safety, while previous studies have also reported higher revision rates and lower implant survivorship after UKA than after TKA, indicating that long-term durability should be considered alongside its short-term perioperative advantages [6, 13, 15, 18, 25].

This study found no significant differences in the occurrence of major systemic complications between UKA and TKA among patients aged ≥ 80 years. This suggests that the advantages of UKA over TKA in terms of surgical invasiveness may be attenuated by increased perioperative vulnerability in elderly patients. This interpretation is consistent with previous studies in various surgical specialties reporting increased perioperative complications and mortality in patients aged ≥ 80 years [41–44]. However, this does not imply that UKA offers no benefits in this population. For example, RBC transfusion was less frequent in the UKA group than in the TKA group among these patients. In addition, previous studies have demonstrated that patients older than 75 years who underwent UKA achieved better functional outcomes, higher Forgotten Joint scores, and earlier recovery than those who underwent TKA [45, 46]. Therefore, UKA remains a reasonable surgical option for appropriately selected elderly patients. Nevertheless, on the basis of our results, surgeons should recognize that UKA may not reduce the risk of in-hospital mortality and major systemic complications requiring additional procedures or interventions relative to TKA in patients aged ≥ 80 years. Careful perioperative risk assessment is warranted when considering UKA in this age group.

The present study had several limitations. First, the DPC database does not provide detailed clinical information, such as the severity of primary diagnoses or comorbidities. Although baseline characteristics were well balanced between the groups after stabilized IPTW, residual confounding related to surgical indication, patient selection, baseline severity of knee disease, surgeon experience, and hospital case volume could not be excluded. Surgeon experience and hospital case volume may influence outcomes following UKA and TKA [38, 47–49]. Second, miscoding of procedures or diagnoses may have occurred. However, prior validation studies have demonstrated high accuracy of coding in the DPC database [34]. Third, given the nature of a nationwide database, variation in perioperative management, intraoperative procedures, and postoperative protocols across institutions may have introduced unmeasured confounding. However, the large sample size enhances the generalizability and provides sufficient statistical power to detect rare but clinically important complications [50]. Fourth, the primary outcome was limited to a composite of postoperative in-hospital death and major systemic complications requiring additional procedures or interventions. These events were selected to ensure high diagnostic specificity and focus on rare but clinically significant outcomes. Thus, complications not requiring additional interventions were not captured. Fifth, outpatient surgeries were excluded from this study because the database covers only inpatient data. Although outpatient knee arthroplasty has been described [51–53], knee arthroplasty is generally performed in inpatient settings in Japan. Sixth, this was an inpatient database, complications occurring after discharge and long-term outcomes, including implant survival and revision rates, could not be assessed. However, because postoperative care and rehabilitation are typically completed during hospitalization in Japan, where hospital stays are generally much longer than those in North America, Australasia, and Europe [54], the inpatient follow-up period was likely sufficient to capture most early complications. Finally, because the DPC database includes only participating acute care hospitals, the possibility of selection bias cannot be excluded. Despite these limitations, this study provides clinically relevant evidence regarding age-stratified risks of major systemic complications following knee arthroplasty.

## Conclusions

The rates of postoperative RBC transfusion were consistently lower following UKA than TKA across age groups. UKA was associated with significantly lower risks of major systemic complications compared with TKA in patients aged ≤ 79 years, whereas no such difference was observed in patients aged ≥ 80 years. However, residual confounding related to surgical indication and patient selection should be considered when interpreting these findings. These results suggest that age should be carefully considered in surgical decision-making, and that in very elderly patients, appropriate perioperative risk assessment remains essential when considering UKA, similar to that for TKA.

## Supplementary Information

Supplementary material 1.

## Data Availability

The datasets used and/or analyzed during the current study are available from the corresponding author on reasonable request.

## Abbreviations

TKA: Total knee arthroplasty
UKA: Unicompartmental knee arthroplasty
DPC: Diagnosis Procedure Combination
ICD-10: International Classification of Diseases 10th Revision
BMI: Body mass index
Respiratory failure: Respiratory failure requiring mechanical ventilation
Gastrointestinal bleeding/perforation: Gastrointestinal bleeding or peptic ulcer perforation
RBC: Red blood cell
IPTW: Inverse probability of treatment weighting
RR: Risk ratio
CI: Confidence interval
