# Supplementary material for: Age-stratified risk of major systemic complications following unicompartmental versus total knee arthroplasty: a nationwide cohort study
Source: Knee Surg Relat Res. 2026 Jul 31;38:35. doi: 10.1186/s43019-026-00330-8 (PMC13428448; doi:10.1186/s43019-026-00330-8)
Supplement: Supplementary file 1 — Supplementary material 1. [file 43019_2026_330_MOESM1_ESM.doc]

**Supplementary Table 1.** Definitions of preoperative comorbidities based on ICD-10 codes.

| Comorbidity | Definition with the ICD-10^a^ code(s) |
| --- | --- |
| Diabetes mellitus | Use of hypoglycemic agents or insulin, with E10 to E14 at admission |
| Dyslipidemia | Use of lipid-lowering drugs, with E78.0 to E78.5 at admission |
| Hypertension | Use of antihypertensive drugs, with I10 to I15 at admission |
| Ischemic heart disease | I20 to I25 at admission |
| Valve disorder | I34 to I37 and I39.0 to I39.4 at admission |
| Atrial fibrillation/flutter | I48 at admission |
| Heart failure | I50 at admission |
| Cerebrovascular disease | I60 to I69 at admission |
| Malignancy | C00 to C97 at admission |
| Hypothyroidism | E03 at admission |
| Hyperthyroidism | E05 at admission |
| Dementia | F00 to F03 and G30 at admission |
| Parkinson’s disease | G20 to G22 at admission |
| Epilepsy | G40 at admission |
| Chronic lung disease | J40 to J47 and J84 at admission |
| Noninfective enteritis | K50 to K52 at admission |
| Hepatic dysfunction | K70 to K77 at admission |
| Renal dysfunction | N00 to N08, N10 to N16, N18, and N19 at admission |

^a^ International Classification of Diseases 10th Revision.

**Supplementary Table 2.** Definitions of postoperative systemic complications.

| Complication | Definition |
| --- | --- |
| Cardiac arrest | Undergoing cardiopulmonary resuscitation or countershock |
| Acute coronary events | Undergoing percutaneous coronary intervention, or coronary artery bypass grafting |
| Heart failure | Undergoing intra-aortic balloon pumping, use of cardiopulmonary support, or administration of vasopressors for more than 3 days, with diagnosis of heart failure (ICD-10^a^ code: I50) as a complication |
| Aortic aneurysm or dissection | Undergoing surgery or percutaneous intervention for aortic aneurysm or dissection |
| Respiratory failure requiring mechanical ventilation | Requiring mechanical ventilation postoperatively |
| Pulmonary embolism | Undergoing pulmonary embolectomy, pulmonary thromboendarterectomy, placement of an inferior vena cava filter, or thrombolysis, with diagnosis of pulmonary embolism (ICD-10 code: I26) as a complication |
| Cerebrovascular events | Undergoing surgery or percutaneous intervention for cerebrovascular events, with diagnosis of cerebrovascular disease (ICD-10 codes: I60 to I64) as a complication |
| Renal failure requiring hemodialysis | Initiation of hemodialysis postoperatively, with diagnosis of renal failure (ICD-10 codes: N17 to N19) |
| Gastrointestinal bleeding or peptic ulcer perforation | Undergoing endoscopic procedures, percutaneous intervention, or surgery for gastrointestinal bleeding or ulcer perforation |

^a^ International Classification of Diseases 10th Revision.
